# Dr. Rollin Thomas Rolph

**Published:** 1912-07

**Authors:** 


Dr. Rollin Thomas Rolph of Chula Vista, Calif., died at the Agnew State Hospital, Calif., May 15, aged 60, of pernicious anaemia. He was a graduate of Buffalo, 1873 and formerly practiced in Dunkirk and Fredonia.

